# Commentary: Consistency of EEG source localization and connectivity estimates

**DOI:** 10.3389/fnins.2018.00147

**Published:** 2018-03-13

**Authors:** Bahar Moezzi, Mitchell R. Goldsworthy

**Affiliations:** ^1^Robinson Research Institute, School of Medicine, University of Adelaide, Adelaide, SA, Australia; ^2^Discipline of Psychiatry, School of Medicine, University of Adelaide, Adelaide, SA, Australia

**Keywords:** electroencephalography, source reconstruction, consistency, effective connectivity, functional connectivity

The integration of information between functionally specialized and widely distributed brain regions (i.e., connectivity) is fundamentally important for cognition (Bressler and Menon, 2010), and can be estimated using electroencephalography (EEG) (Horwitz, 2003). However, the interpretation of connectivity measures from sensor-level EEG recordings is not straightforward. Instead, the neuroanatomical sources of recorded sensor-level data can be reconstructed by first finding the scalp potentials that result from hypothetical current distributions inside the head (i.e., the forward problem), then applying this to the actual EEG data to estimate back the sources that fit the measurements (i.e., the inverse problem). Several publicly available software packages have been developed for analysis of source-level EEG data. There exist many different models for solving the forward and inverse problems, and these can be implemented using several analysis packages. For source-level EEG to be a reliable tool for measuring connectivity, source estimates should be consistent across these different combinations of commonly used model parameters and software packages. If not, this would have important implications for reproducibility of EEG studies reconstructing brain activation and connectivity from neuronal sources.

Recently, Mahjoory et al. (2017) investigated the consistency of source localization and connectivity estimates across a host of widely used analysis pipelines. They tested 14 pipelines consisting of different combinations of (1) software packages including Brainstorm, FieldTrip and Berlin Toolbox; (2) inverse models including weighted minimum-norm estimate (WMNE), exact low resolution electromagnetic tomography (eLORETA) and linearly constrained minimum-variance (LCMV); and (3) forward models including boundary element, finite element and spherical harmonics expansions methods. Resting EEG data (eyes closed) was recorded from 65 healthy subjects as part of two different experiments; one on attentional processes (Fasor data) and the other as part of a brain-computer interface study (Würzburg data). In the latter, two sessions on separate days were conducted per subject. Source localization and connectivity estimates were computed for alpha-band (8–13 Hz) oscillations. Connectivity measures included imaginary coherence (Nolte et al., 2004), reflecting temporal correlations of neural activity between brain areas (i.e., functional connectivity), and phase slope index (Nolte et al., 2008), reflecting directed interactions (i.e. effective connectivity). To evaluate consistency across pipelines they computed grand-average correlations between localization or connectivity results for all pairs of pipelines. To evaluate between-study consistency they computed grand-average correlations between results of the two experiments for each pipeline. To evaluate within-subjects consistency they computed correlation between the two sessions of the Würzburg experiment for each subject and pipeline then averaged across subjects. To evaluate between-subjects consistency they computed correlations between datasets of distinct subjects separately for the Fasor and Würzburg experiments then averaged across pairs of subjects.

Mahjoory et al. (2017) showed that: connectivity patterns between EEG electrodes vary depending on the choice of electrical reference, supporting the use of source reconstruction for connectivity analyses. Source localization estimates, mapped onto cortical surface, have a smaller maximum and are more focally concentrated in the occipital region when LCMV is used than when eLORETA or WMNE is used. Patterns of interactions estimated from the reconstructed sources vary when different inverse methods are used. Across all pipelines source localization estimates are more consistent than functional connectivity estimates, followed by effective connectivity estimates. Average correlation across different combinations of forward models is higher than when varying inverse methods or software packages. For source localization and connectivity, correlation between WMNE and eLORETA based estimates exceeds correlation between LCMV and either eLORETA or WMNE based estimates. Source localization and connectivity results are most consistent between-studies, followed by within-subjects and then between-subjects. Between-study, within-subjects and between-subjects consistencies are highest for source localization estimates followed by functional and then effective connectivity estimates. Between-study, within-subjects and between-subjects consistencies, computed at the sensor-level, are in general similar to the average source-level results.

There are several important issues to bear in mind when considering the consistency of EEG measures in the source-space, not least of which is the physiological state of participants at the time of recording. The resting condition is a challenging state for source-level analysis, and likely represents a major source of inconsistency in the source localization and connectivity outcomes. Related to this is the arousal state of the subjects. This has been shown to affect physiological significance of source localization and alter connectivity estimates using various methods and modalities (Kaufmann et al., 2005; Massimini et al., 2005; Murphy et al., 2009; Ventouras et al., 2010). Therefore particularly important for within-subjects and between-subjects analysis, variability in the subjects' arousal state likely account for some of the variability in the estimates.

Based on the findings of Mahjoory et al. (2017), we highlight the importance of utilizing a priori assumptions based on physiological information determined using other modalities. Magnetoencephalography (MEG) and functional magnetic resonance imaging (fMRI) are non-invasive recording techniques used to study human brain activity. It is suggested that combining EEG with these modalities may produce more accurate source localization estimates than either modality alone (Liu et al., 2002; Groening et al., 2009), presumably improving the consistency of source localization and connectivity estimates. Additionally, transcranial magnetic stimulation (TMS) is a technique that enables non-invasive manipulation of neural activity. TMS combined with EEG can be used to provide priors for EEG connectivity analysis and validate the connectivity results (Bortoletto et al., 2015).

There are several limitations of Mahjoory et al. (2017), many of which were addressed in the original work. However, one additional point to consider is the statistical approach for assessing consistency. In Mahjoory et al. (2017) consistency is quantified by reporting Pearson correlation coefficient. However, other measures may be suited to estimate consistency, such as intraclass correlation coefficient or the standard error of measurement (Bédard et al., 2000).

Mahjoory et al. (2017) presented the first comprehensive assessment of consistency of EEG source localization and connectivity estimates across widely used forward and inverse methods. Their study is an important contribution toward a consensus about source-level methodologies. Their findings highlight the need to take caution when interpreting source-level outcomes, particularly in clinical settings. However, this should not discourage researchers from studying EEG recordings in the source-space.

## Author contributions

All authors listed have made a substantial, direct and intellectual contribution to the work, and approved it for publication.

### Conflict of interest statement

The authors declare that the research was conducted in the absence of any commercial or financial relationships that could be construed as a potential conflict of interest.
